# Herbicide-resistant cotton (*Gossypium hirsutum*) plants: an alternative way of manual weed removal

**DOI:** 10.1186/s13104-015-1397-0

**Published:** 2015-09-17

**Authors:** Ayesha Latif, Abdul Qayyum Rao, Muhammad Azmat Ullah Khan, Naila Shahid, Kamran Shehzad Bajwa, Muhammad Aleem Ashraf, Malik Adil Abbas, Muhammad Azam, Ahmad Ali Shahid, Idrees Ahmad Nasir, Tayyab Husnain

**Affiliations:** CEMB University of the Punjab, 87 West canal Bank Road, Lahore, 53700 Pakistan

**Keywords:** Glyphosate, EPSPS gene, Stable transformation, Cotton, Weeds

## Abstract

**Background:**

Cotton yield has been badly affected by different insects and weed competition. In Past Application of multiple chemicals is required to manage insects and weed control was achieved by different conventional means, such as hand weeding, crop rotation and polyculture, because no synthetic chemicals were available. The control methods shifted towards high input and target-oriented methods after the discovery of synthetic herbicide in the 1930s. To utilise the transgenic approach, cotton plants expressing the codon-optimised CEMB GTGene were produced in the present study.

**Results:**

Local cotton variety CEMB-02 containing Cry1Ac and Cry2A in single cassette was transformed by synthetic codon-optimised 5-enolpyruvylshikimate-3-phosphate synthase gene cloned into pCAMBIA 1301 vector under 35S promoter with *Agrobacterium tumifaciens*. Putative transgenic plants were screened in MS medium containing 120 µmol/L glyphosate. Integration and expression of the gene were evaluated by PCR from genomic DNA and ELISA from protein. A 1.4-kb PCR product for Glyphosate and 167-bp product for Cry2A were obtained by amplification through gene specific primers. Expression level of Glyphosate and Bt proteins in two transgenic lines were recorded to be 0.362, 0.325 µg/g leaf and 0.390, 0.300 µg/g leaf respectively. FISH analysis of transgenic lines demonstrates the presence of one and two copy no. of Cp4 EPSPS transgene respectively. Efficacy of the transgene Cp4 EPSPS was further evaluated by Glyphosate spray (41 %) assay at 1900 ml/acre and insect bioassay which shows 100 %mortality of insect feeding on transgenic lines as compared to control.

**Conclusion:**

The present study shows that the transgenic lines produced in this study were resistant not only to insects but also equally good against 1900 ml/acre field spray concentration of glyphosate.

**Electronic supplementary material:**

The online version of this article (doi:10.1186/s13104-015-1397-0) contains supplementary material, which is available to authorized users.

## Background

Glyphosate [*N*-(phosphonomethyl) glycine] is a nonselective foliar-applied herbicide that provides cheap control options for annual, perennial, and biennial herbaceous species of grasses, sedges, and broad leaf weeds, as well as woody brush and tree species, and has been used for over several decades [1, 2]. Glyphosate inhibits 5-enolpyruvyls-Shikimate-3-phosphate (EPSP) synthase [3]. The formation of EPSP from the enolpyruvyl moiety of phosphoenolpyruvate (PEP) and shikim-ate-3-phosphate (S3P) is catalysed by this enzyme, which is a key step in the production of the aromatic acids phenylalanine, tryptophan and tyrosine along with many important secondary compounds, such as indole acetic acid, lignin and phytoalexins [4]. The enzyme 5-enolpyruvylshikimate-3-phosphate synthase (EPSPS) and its pathway are distinctive to plants and microbes and are nontoxic to animals. This mode of action accounts for all commercial glyphosate-tolerant crops. Therefore, glyphosate has become the world’s most valuable agrochemical due to its low cost, low toxicity, effective broad-spectrum weed control and availability of transgenic crop tolerance.

Application of multiple chemicals is required to manage insect and weed control throughout the cotton-growing season. The most important management practices in field crops are weed control. Cotton yield has been badly affected by the crop weed competition during the first few weeks (4–7) after planting [5].

Glyphosate-resistant cotton was first commercialised in 1997, and the acreage was dramatically increased afterward. In China, Xie et al. [6] obtained 65 regenerated plants using the hypocotyl of CRI 35 as an explant Zhao et al. [7] introduced aroAM12; a glyphosate-resistant gene encoded for 5-enolpyruvyl-shikimate-3-phosphate synthase (EPSPS), combined with an insect-resistant gene, Bts1m, into Shiyuan-321 using an Agrobacterium-mediated method and obtained 52 regenerated plants. Molecular analysis revealed that 38 of these plants had both the aroimAM12 and Bts1m genes. Liu et al. [8] obtained 3 glyphosate-resistance transgenic plants using the pollen-tube pathway transformation technique. The Cp4 EPSPS gene was introduced into the GTS 40-3-2 soybean line using a biolistic gun by Padgette et al. [9], but this event did not significantly alter soybean morphology or agronomic characteristics (such as time to flowering and pod set). Efforts to develop GR corn began in the late 1980s. The first commercial event was GA21, which relies on maize EPSPS genes (zm-EPSPS) that were modified to be glyphosate-insensitive [10].

In an effort to develop cotton with an enhanced insect and herbicide resistance, this study was performed to introduce cp4EPSPS gene, which promotes glyphosate resistance, into insect-resistant transgenic cotton using an Agrobacterium-mediated transformation. Here, we report our successful recovery of transgenic cotton plants expressing cp4EPSPS transgenes in insect-resistant cotton genotypes.

## Results

### Construction of the plant expression vector: pCambia 1301 cp4EPSPS

**Fig. 1:**
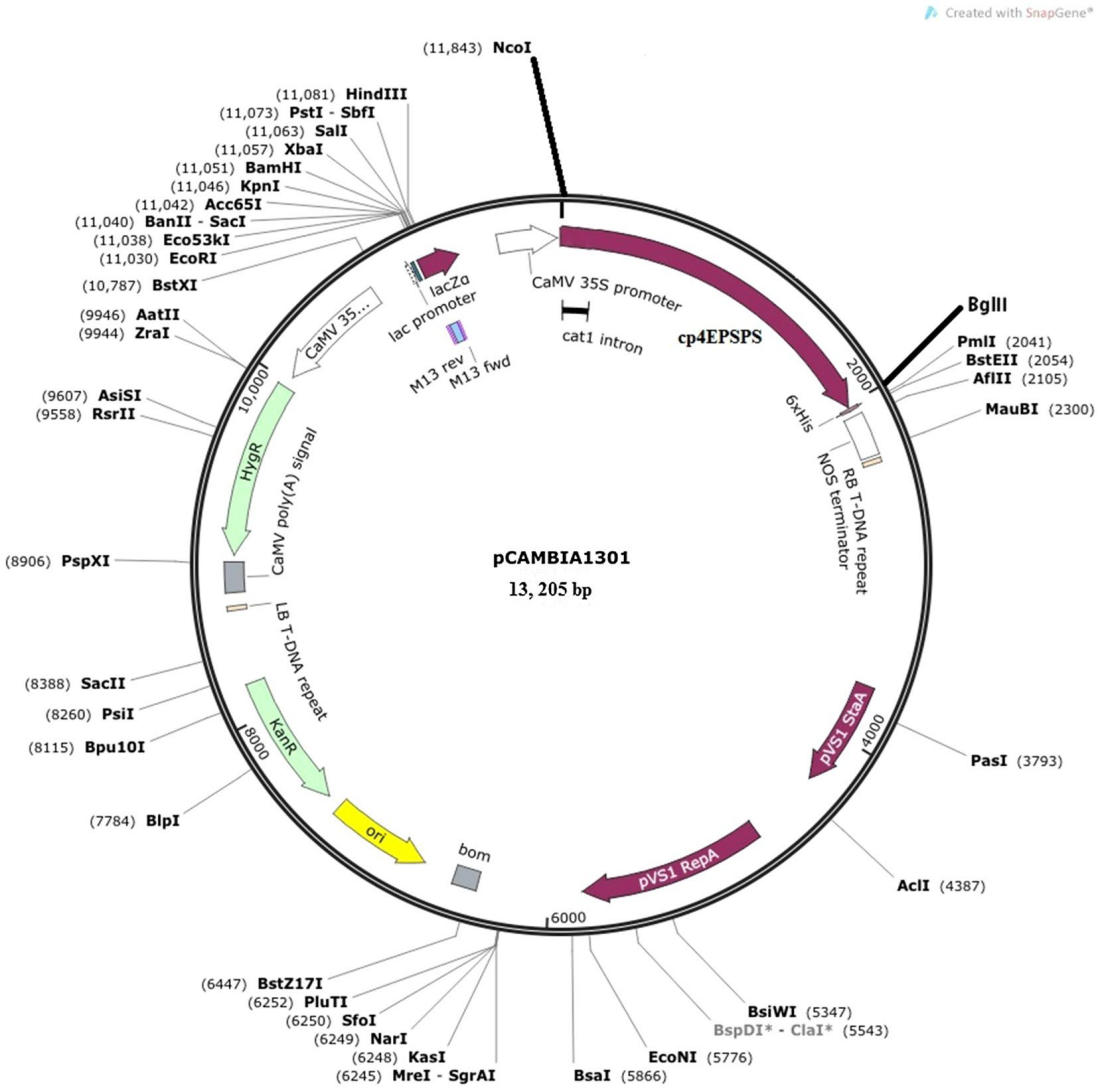
Construct map of codon optimized cp4EPSPS cloned gene in pCAMBIA 1301 vector

The pCambia 1301 cp4EPSPS construct (Fig. 1) was developed by amplification with gene-specific primers with specific NCOI and BglII digestion sites. Digestion of both the insert and vector produced sticky ends. Ligation of the 1368-bp insert into the 11,837-bp vector generated a 13,205-bp ligated product, which was confirmed by PCR amplification using gene-specific and vector primers as well as through restriction digestion with NCOI and BglII that generated 1.4-kb insert and 11 kb vector products [11].

### Agrobacterium-mediated transformation of CEMB-02 with pCambia 1301 cp4EPSPS

To incorporate the cp4EPSPS gene into CEMB-02, Agrobacterium-mediated transformation was used. The cotton seed surface was sterilized by 1 g/l HgCl2 and 1 g/l of 10 % sodium dodecyl sulfate (SDS). Mature embryos were isolated from germinating seeds and a cut was made at the apex of the shoot with a sterilized blade. Afterwards, the embryos were co-cultivated for 1 h with an Agrobacterium strain containing cp4EPSPS gene. The embryos were then dried on filter paper and cultured on MS medium for 3 days at 28 °C. After 3 days, embryos were subcultured on MS medium containing 120 µmol/L glyphosate optimized for cotton plants for selection. At 120 µmol/L, control plants in this study died whereas putative transgenic plants survived. A hundred putative transgenic plants out of 10,000 transformed embryos were obtained after 8 weeks of selection. The putative transgenic plants were shifted to soil containing sand, silt and clay in the ratio called loamy soil, in pots. The stable putative transgenic plants were subjected to molecular analysis after 15–20 days following shifting [11, 12].

### Molecular analyses of putative transgenic plants

The plants that survived that glyphosate selection successfully, transferred to the soil in pots, were considered to be putative transgenics. These plants were further analysed for the presence and expression of the cp4EPSPS gene (Additional file 1: Table S2).

### Detection of the cp4EPSPS gene in Cotton plants

**Fig. 2:**
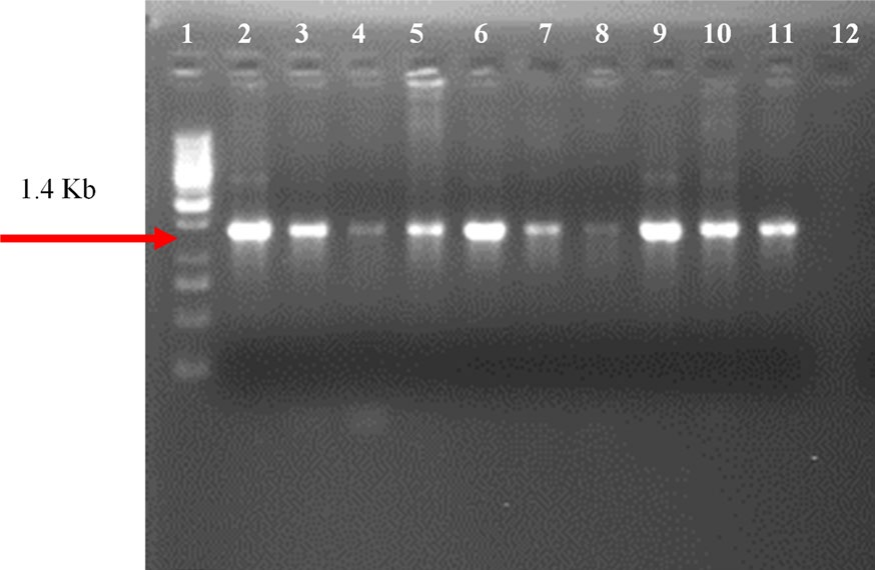
Confirmation of transgene in Cotton by PCR. *Lane 1* 1 Kb ladder (thermoscientific, cat #SM0312). *Lane 2* Positive control. *Lanes 3*–*11* Positive transgenic plants (CEMB 1330-4, CEMB 1330-12, CEMB 1330-17, CEMB 1330-21, CEMB 1317-4, CEMB 1317-5, CEMB 1317-9, CEMB 1317-15 and CEMB 1317-19 respectively). *Lane 12* Negative control

Insertion of the cp4EPSPS gene into the cotton plants was detected by PCR and 1.4-kb fragment was amplified with gene-specific primers. Nine of 27 plants, CEMB 1330-4, CEMB 1330-12, CEMB 1330-17, CEMB 1330-21, CEMB 1317-4, CEMB 1317-5, CEMB 1317-9, CEMB 1317-15 and CEMB 1317-19, were found positive transgenic cotton plants with no amplification in the negative control (CIM-482) plant in all three generations (Fig. 2). The transgenic plants were also evaluated for the presence of Cry1Ac genes by using gene specific primers. Amplification of 468 bp was obtained on 1 % agarose gel. The possiblity of *Agrobaterium* contamination was removed by complete absence of amplified product with *Vir G* primers as done by [11–13]. T0 progeny analysis have been sown by Awan et al. [11].

### Expression of the cp4EPSPS gene in Cotton Plants

The plants that were positive in the PCR assay were further analysed for protein expression of the cp4EPSPS gene. Total protein was isolated from nine PCR-confirmed transgenic plants and analysed by an ELISA. The transgenic plants, CEMB 1330-4, CEMB 1330-12, CEMB 1330-17, CEMB 1330-21, CEMB 1317-4, CEMB 1317-5, CEMB 1317-9, CEMB 1317-15 and CEMB 1317-19, expressed the cp4EPSPS gene protein, as detected by ELISA, while no expression was observed in negative control plants in all three generations (Additional file 1: Table S2).

### Agronomic and fibre traits of Transgenic Plants

The agronomic traits of the transgenic plants were noted to be i.e., plant height 160–180 cm, sympodia 15–25, monopodia 0, number of bolls per plant 90–120, average weight of the boll 3.5 g. The fibre trait were as GOT 41 %, Micronare 4.34, maturity 0.87, upper half mean length 1.39, uniformity index 82.9 % and strength 30.8 g/tex.

### Determination of the copy number of the transgene

**Fig. 3:**
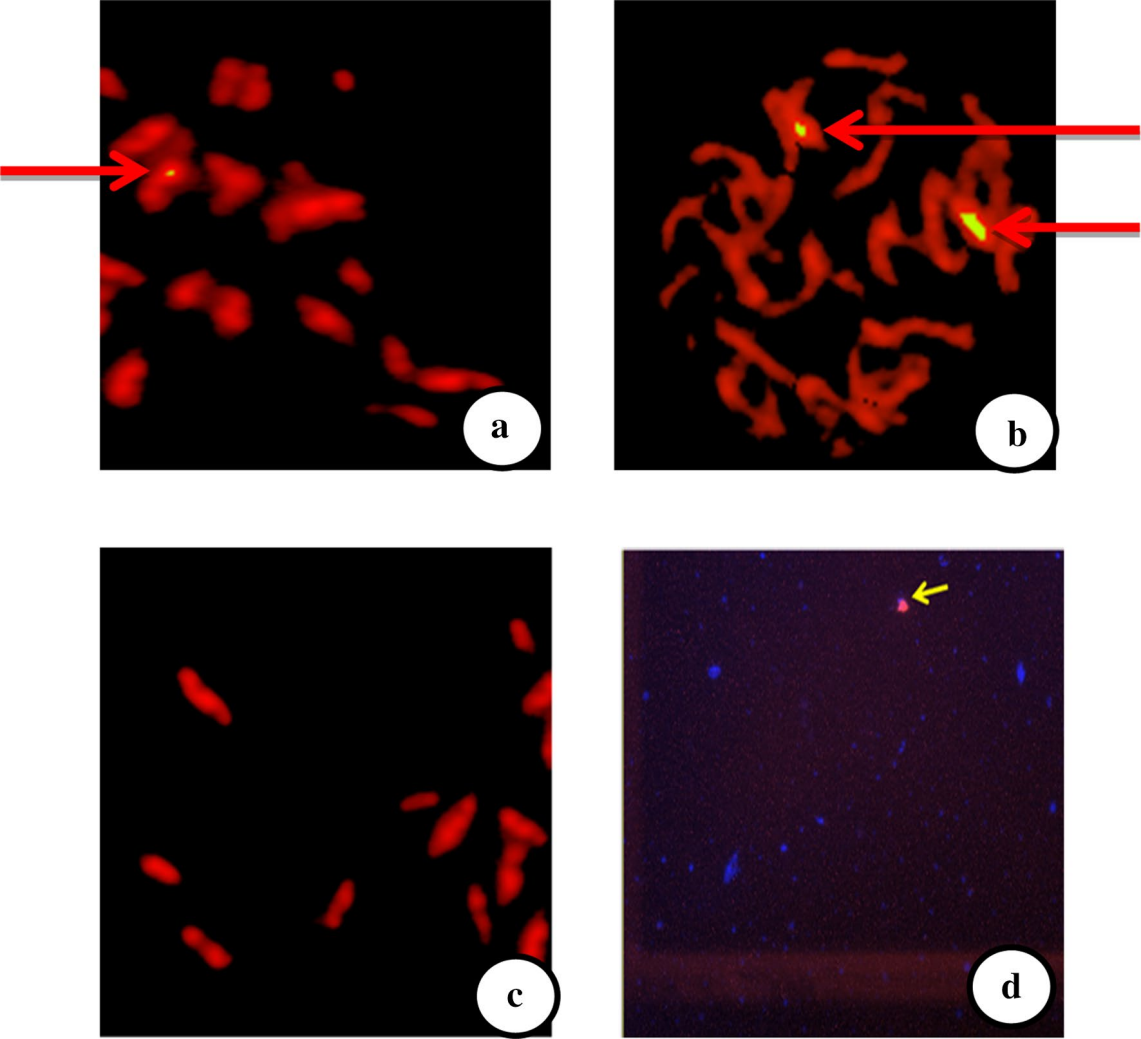
FISH analysis for GT transgenic cotton plants. **a** Transgenic line CEMB-1317-15 showing one copy of the transgene whereas in **b** transgenic line CEMB-1330-4 showed two copies of transgene while **c** is the negative control plant which is without signal of hybridization probe. **d** positive control transgenic plant

All GTGene-transformed plants, confirmed by PCR, were analysed by fluorescence in situ hybridisation (FISH) to determine the transgene copy number. A visible signal was obtained after hybridisation with a fluorescently labelled gene-specific probe, as shown in Fig. 3, and no signal was obtained from the non-transgenic control plants. The variation in copy number of the GTGene was observed in different transgenic plant lines. The transgenic line CEMB-1317-15 had one copy of the transgene (Fig. 3a), while two copies of the transgene were observed in the transgenic line CEMB-1330-4 (Fig. 3b). The control plants (CIM-482 variety) had no FISH signal (Fig. 3c). The variation in expression could be due to difference in copy number of transgene as studied by Rao et al. [19]

### Glyphosate spray assay

**Fig. 4:**
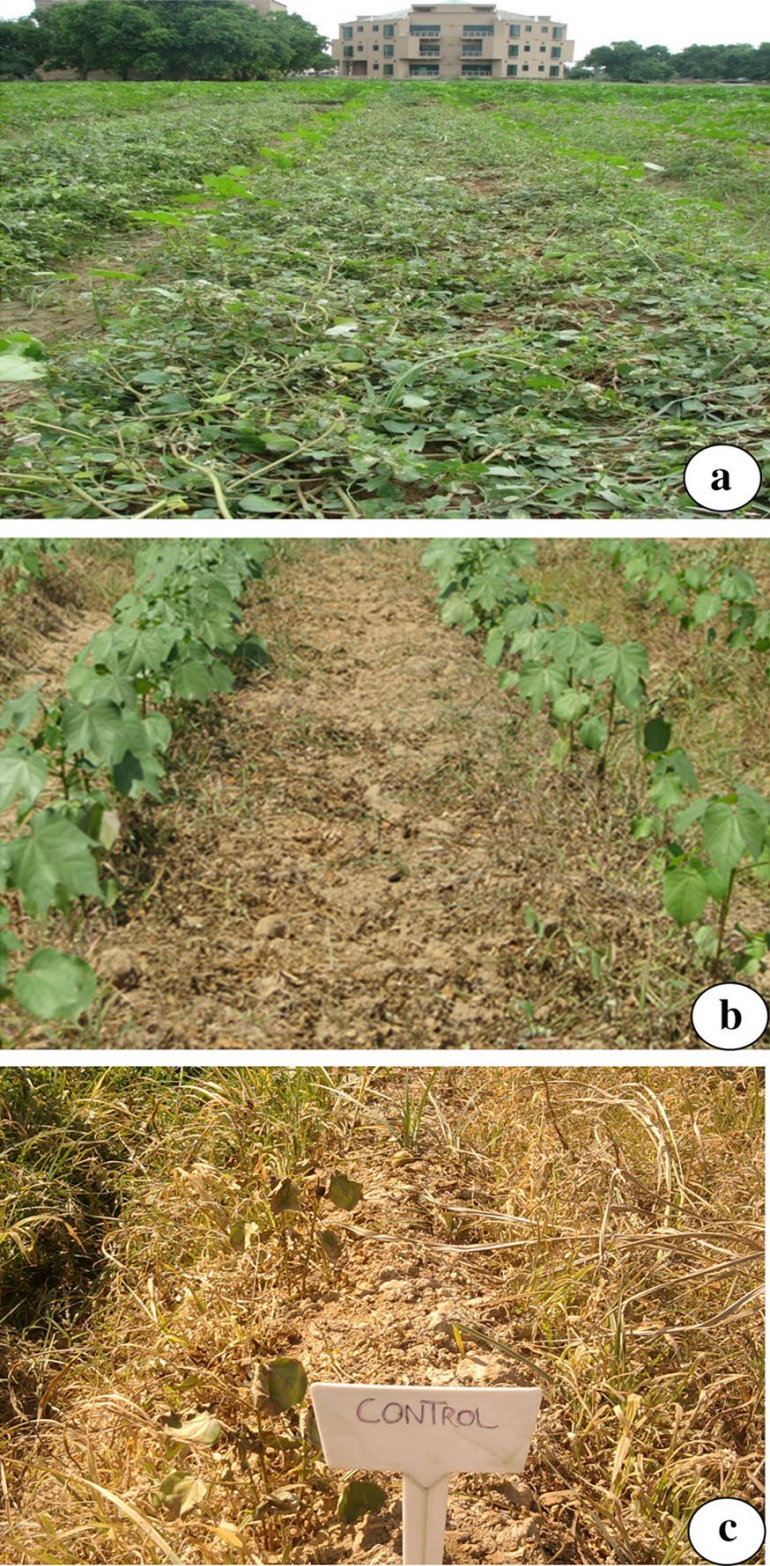
Spray of glyphosate on GTG transgenic plant (1900 ml/acre). **a** Field after 3 days of spray where most the weeds have started to turn yellow. **b** Weeds died in Field after 5 days of spray (**c**) negative control plant along with weeds which shows chlorotic and wilted appearance

The efficacy of the transgenes in the transgenic plants was further confirmed by the leaf glyphosate spray assay, as shown in Fig. 4. The results showed that the prevalent weeds *Euphorbia helioscopia, Euphorbia prostrate, Cynodon dactylon, Cyperus rotundus, Cyperus dactylon, Portulaca oleracea, Sorghum halepense, Trianthema portulacastrum, Amaranthus viridis, Echinochloa colon, Setaria viridis, Corchorus tridens, Digera muricata* and *Tribulus terrestris* were killed by a spray a glyphosate 1900 ml/acre (glyphosate 41 % w/w) in all three generations whereas the glyphosate tolerance gene transgenic plant survived at this concentration of the glyphosate (Fig. 4).

## Conclusions

Weeds are unwanted plants that comprise approximately 0.1 % of the agro system world flora. Weeds act as major factor for the declining crop yield by competing for resources, such as water, light and nutrients. Approximately 30 % of yield losses in cotton are caused by weeds [14]. Therefore, the control of weeds remains a major concern for crop producers. In the past, weed control was achieved by different conventional means, such as hand weeding, crop rotation and polyculture, because no synthetic chemicals were available. The control methods shifted towards high input and target-oriented methods after the discovery of synthetic herbicide in the 1930s. To utilise the transgenic approach, cotton plants expressing the codon-optimised CEMB GTGene were produced. Codon optimization accordin to *G. hirsutum* was performed to achieve the maximum expression of the transgene. Cloning of the codon-optimized CEMB cp4EPSPS gene (GTGene) was performed with a restriction digestion with the NCOI and BglII enzymes in pCAMBIA 1301 according to Kiani et al. [23]. Agrobacterium-inoculation of the mature cotton embryos after injury was [20, 23]. The plantlets were moved to pots and then to a greenhouse after successful screening with a marker gene and confirmation by PCR according to Bajwa et al. [20]. Amplification of 1.4 kb of the cp4EPSPS gene as shown in Fig. 2 by gene-specific primers confirmed the successful transformation of cp4EPSPS gene in Bt-transgenic cotton lines to make these cotton lines resistant to broad-spectrum glyphosate and insects, respectively, as done by Rao et al. [24] to overexpress Phytochrome B gene in cotton for physiological improvement. Similarly absence of amplified product by VirG primers ensures the successful integration of transgene in plants and eliminates the confusion of Agrobacterium contamination as done by Bajwa et al. [12]. Moreover, these confirmed glyphosate-tolerant plants were further evaluated for integration of the CryIIA insect-resistant gene. Amplification of the 468-bp fragment of CryIIA gene-specific primers verified successful integration of the CryIIA insect-resistant gene in two lines of the cotton variety CIM 482 which was named CEMB-02 [15, 16]. Expression of the transgene in plants is vital for successful development of the transgenic product. Cp4EPSPS expression in transgenic plants was evaluated by ELISA according to Kiani et al. [17] using the Envirologic kit (cat #AP010). The expression of the Bt protein was also evaluated using the ELISA Envirologix kit for confirmation of all transgenic proteins in the two transgenic lines of CEMB (Additional file 1: Table S3). Figure 3a, b depicts the copy number of the cp4EPSPS gene in the two transgenic lines CEMB-1317-15 and CEMB-1330-4. The results clearly demonstrated that one copy number of the transgene cp4EPSPS was identified in CEMB-1317-15, while CEMB-1330-4 showed two copy numbers, as indicated by the FISH signals, but no signal was observed in the negative-control, non-transgenic plant (Fig. 3c). These results correlated with the findings of [18, 19] in which the same signals of the transgene were identified in different transgenic plants using the same methodology of transformation and in situ hybridisation techniques. Moreover, the expression of the transgene cp4EPSPS and the integrated Bt gene was further evaluated using the insect assay and the spray assay as performed previously by Bakhsh et al. [20]. Figure 4 showed the results of three generation studies for field spray assays with 1900 ml/acre of glyphosate that further confirmed the efficacy of the transgenes in two cotton lines.

## Methods

### Codon optimisation

The native cp4 EPSPS gene was analysed for differences in codon bias and was optimised according to the codon preference of the cotton plant. The codon usage table of cotton in the codon usage database (http://www.Kazusa.or.jp/codon) was downloaded and modified (Additional file 1: Table S1) to eliminate those codons having less than 10 percent preference, and such codons were proportionately replaced by their synonymous codons according to their weight [21].

### Cloning of the cp4 EPSPS gene into pCAMBIA 1301

A transgenic cassette was created for dicot expression from a combination of the promoter sequence, cp4 EPSPS, and transcriptional termination sequences. A kanamycin selectable marker (neomycin phosphotransferase II, nptII) was used for maintenance in bacteria. The cauliflower mosaic virus promoter 35S [22] was used as a promoter in the cassette. Restriction digestion of the pCambia 1301 vector was performed using the restriction enzymes NCO1 and BglII. The restriction site was created in cp4 EPSPS by amplification with primers containing the sequence of restriction enzymes. The eluted product was ligated with the pCambia 1301 vector using the Fermentas ligation kit (Cat #EL0014). To confirm the cloning, the digestion procedure was performed using the restriction enzymes listed above. Digestion reactions were performed at 37 °C overnight in a PCR machine. Each 20-µl reaction contained 4–5 µg DNA, 2 µl 10 × recommended restriction buffer, and 5 U of NCO1 and BglII (Fermentas, USA). The DNA digestion was visually inspected under the UV light after agarose gel (1.5 %) electrophoresis. Orientation of the cp4 EPSPS gene was confirmed using the vector Forward primer GATTGATGTGATATCTCCACT and the gene Reverse primer ACTCTACCCATAGGTCTCTTAG and by sequencing with gene-specific primers.

### Plant material and plant transformation

Two Bt transgenic lines, CEMB-1317 and CEMB-1330, were selected for the transformation of the codon-optimised synthetic cp4 EPSPS (glyphosate tolerant) gene according to their Bt expression and yield potential. Electroporation of the cp4 EPSPS gene construct into the *Agrobacterium* strain LBA4404 and its transformation into two Bt transgenic cotton lines using *Agrobacterium* was performed according to the procedure described by Rao et al. [23].

### Confirmation of the transgene cp4EPSPS in the Bt transgenic cotton lines

To confirm the presence of the transgene cp4 EPSPS in Bt-transgenic cotton lines, amplification of a 1.4 kb-fragment of the glyphosate-resistant gene was amplified by a PCR according to Cronn et al. [24] at the T0 [11], T1 and T2 stages. Gene-specific primers used for amplification in this study were: Forward Primer CATGCCATGGATGTCCCACGGTGCTT and reverse Primer TCTCGGAGATCTCTAAGCAGCCTTAGTGTC. The reaction was performed as follows: denaturation for 3 min at 94 °C, annealing for 1 min at 60 °C, and extension for 1.20 min at 72 °C and a final extension for 10 min at 72 °C. The total number of cycles for this reaction was 40. Moreover to confirm the integration of transgene cp4EPSPS in transgenic plants and to rectify the confusion of *Agrobacterium* contamination the transgenic plants were also confirmed through Vir G gene primers of *Agrobaterium* as done by Bajwa et al. [13].

### Enzyme-linked immunosorbant assay (ELISA) of transgenic plants

The PCR-verified cp4 EPSPS gene transgenic plants were analysed by an enzyme-linked immunosorbant assay (ELISA) for protein expression. Protein was isolated from the fresh leaves of the plant with a protein extraction buffer at the T0, T1 and T2 stages using an Envirologix ELISA Kit (Cat # AP010). Total crude protein was quantified by the Bradford assay (Bradford 1976). For each sample, 20 µg of crude protein were loaded into each well of an ELISA-kit quali plate, and ELISA was performed according to the manufacturer’s instructions [25].

### Fluorescence in situ hybridisation (FISH)

Fluorescence in situ hybridisation (FISH) of the transgenic plants was performed according to the procedure described by [18]. Probe was labelled by Mirus Label IT^®^ FISH Cy3 Kit (cat# MIR6510, MJS BioLynx Inc. P.O Bag 1150, 300 Laurier Blvd. Brockville, ON K6 V 5W1, Canada). Chromosmes was were prepared from the growing root tips and hybridized with probe. The signals were detected fluorescent signals were detected by Fluorescent microscope (Carl Zeiss AXIO 100) using appropriate filter set. The picture of fluorescence signal was taken by CCD camera attached with microscope and analyzed by using software Genus 3.7 provided by Cytovision Applied Imaging Systems. The karyotyping was done using the same software package.

### Glyphosate spray assay

Transgenic cotton lines were initially selected by the greenhouse spray assay. Seeds were acquired from T0-transformed plants (called T1 Plants) that demonstrated good resistance to glyphosate at the rate of 1100 ml/ha but sometimes control plants also survive also complete weed removal does not take place at this dose hence T1- and T2-generation cotton lines were selected for screening using the spray test. The transformed plants were planted in the field and treated with up to 1900 ml/ha of Roundup-Ready glyphosate (field-equivalent rate) to determine which plants possessed the greatest degree of tolerance. The same amount of glyphosate spray was also added to non-transformed plants, which were used as a control. The vegetative injury level of both transformed and a control plant was recorded. The most tolerant transgenic lines were utilised for tolerance comparisons [26] (Additional files 2, 3, 4).

## Additional files

**Additional file 1.** Transformation efficieny and Molecular analysis of putative transgenic crop.
**Additional file 2.** Comparison of CEMB glyphosate resistant gene vs monsanto glyphosate resistant gene (1/3).
**Additional file 3.** Comparison of CEMB glyphosate resistant gene vs monsanto glyphosate resistant gene (2/3).
**Additional file 4.** Comparison of CEMB glyphosate resistant gene vs monsanto glyphosate resistant gene (3/3).

## Abbreviations

ELISA: enzyme linked immunosorbent assay
PCR: polymerase chain reaction
EPSPS: 5-enolpyruvylshikimate-3-phosphate synthase
FISH: fluorescent in situ hybridization
GTG: glyphosate tolerant gene
