# Supplementary material for: Herbicide-resistant cotton (Gossypium hirsutum) plants: an alternative way of manual weed removal
Source: BMC Res Notes. 2015 Sep 17;8:453. doi: 10.1186/s13104-015-1397-0 (PMC4574545; doi:10.1186/s13104-015-1397-0)
Supplement: Supplementary file 1 — Additional file 1. Transformation efficieny and Molecular analysis of putative transgenic crop. [file 13104_2015_1397_MOESM1_ESM.doc]

| **Table S1: Transformation efficiencies of Cotton Plants** | | | | | |
| --- | --- | --- | --- | --- | --- |
|  |  |  | nPlants | |  |
| No | nTotal | nControl | Control | Transgenic | T.E. % |
| 1 | 1000 | 50 | 41 | 7 | 0.73 |
| 2 | 1000 | 50 | 35 | 9 | 0.94 |
| 3 | 1000 | 50 | 33 | 15 | 1.55 |
| 4 | 1000 | 50 | 39 | 11 | 1.2 |
| 5 | 1000 | 50 | 32 | 8 | 0.84 |
| 6 | 1000 | 50 | 23 | 12 | 1.26 |
| 7 | 1000 | 50 | 37 | 10 | 1.05 |
| Total | 7000 | 350 | 250 | 72 | 1.02 |

| **Table S2: The molecular analysis included PCR, ELISA and FISH** | | | |
| --- | --- | --- | --- |
| **Plants** | **PCR** | **Southern Blot** | **ELISA** |
| **CEMB 1330-4** | **+** | **+** | **+** |
| **CEMB 1330-12** | **+** | **+** | **+** |
| **CEMB 1330-17** | **+** | **+** | **+** |
| **CEMB 1330-21** | **+** | **+** | **+** |
| **CEMB 1317-4** | **+** | **+** | **+** |
| **CEMB 1317-5** | **+** | **+** | **+** |
| **CEMB 1317-9** | **+** | **+** | **+** |
| **CEMB 1317-15** | **+** | **+** | **+** |
| **CEMB 1317-19** | **+** | **+** | **+** |
| **CEMB 22** | **+** | **+** | **+Ve Control** |
| **CIM-482** | **-** | **-** | **-Ve Control** |

**Table S3: Quantification of Cp4EPSPS protein through ELISA**

| **Plants** | **Protein Quantity (µg/g)** |
| --- | --- |
| CEMB 1330-4 | 1.810 |
| CEMB 1330-12 | 1.646 |
| CEMB 1330-17 | 1.723 |
| CEMB 1330-21 | 1.662 |
| CEMB 1317-4 | 1.692 |
| CEMB 1317-5 | 1.860 |
| CEMB 1317-9 | 1.767 |
| CEMB 1317-15 | 2.08 |
| CEMB 1317-19 | 1.723 |
| CEMB 22 (+Ve Control) | 1.921 |
| CIM-482 (-Ve Control) | 0.000 |
