# Supplementary figures and images for: Herbicide-resistant cotton (Gossypium hirsutum) plants: an alternative way of manual weed removal
Source: BMC Res Notes. 2015 Sep 17;8:453. doi: 10.1186/s13104-015-1397-0 (PMC4574545; doi:10.1186/s13104-015-1397-0)

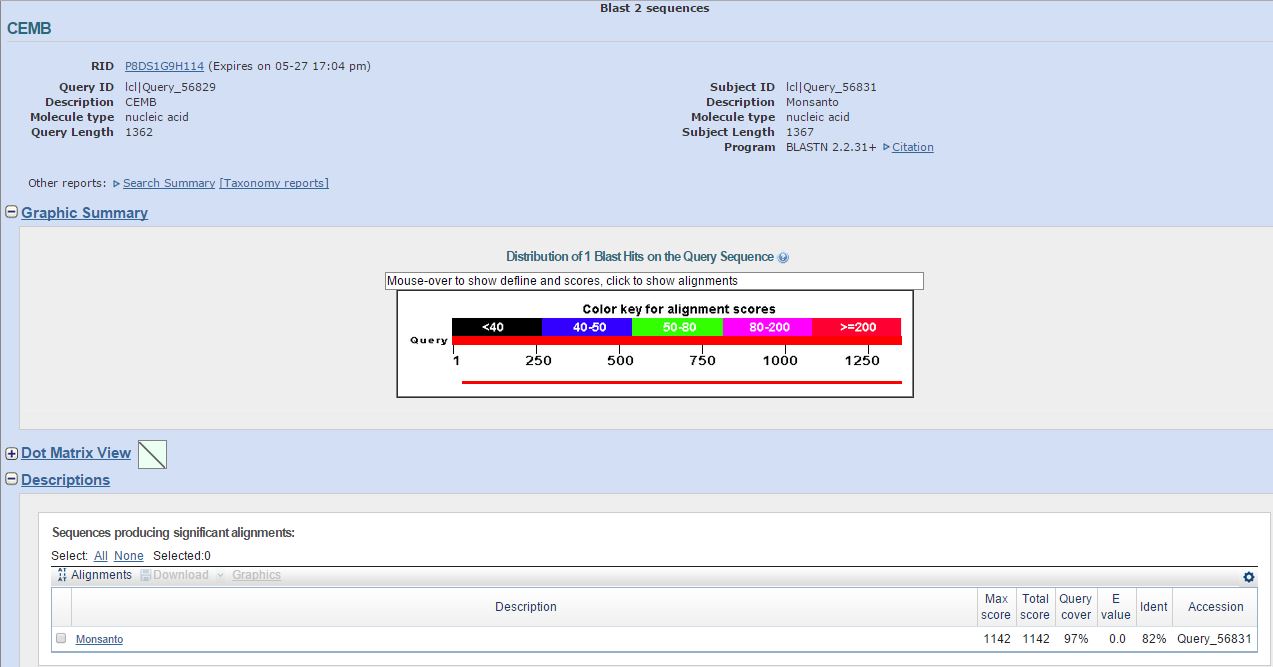

Supplement: Supplementary file 2 — Additional file 2. Comparison of CEMB glyphosate resistant gene vs monsanto glyphosate resistant gene (1/3). [file 13104_2015_1397_MOESM2_ESM.jpeg]

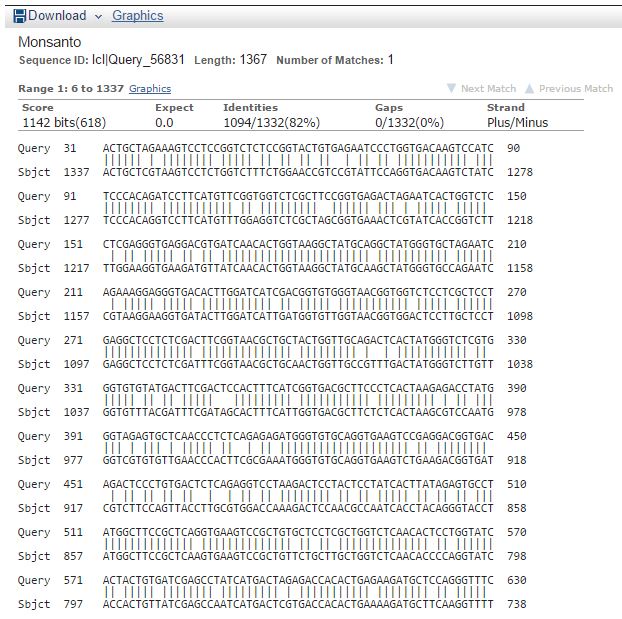

Supplement: Supplementary file 3 — Additional file 3. Comparison of CEMB glyphosate resistant gene vs monsanto glyphosate resistant gene (2/3). [file 13104_2015_1397_MOESM3_ESM.jpeg]

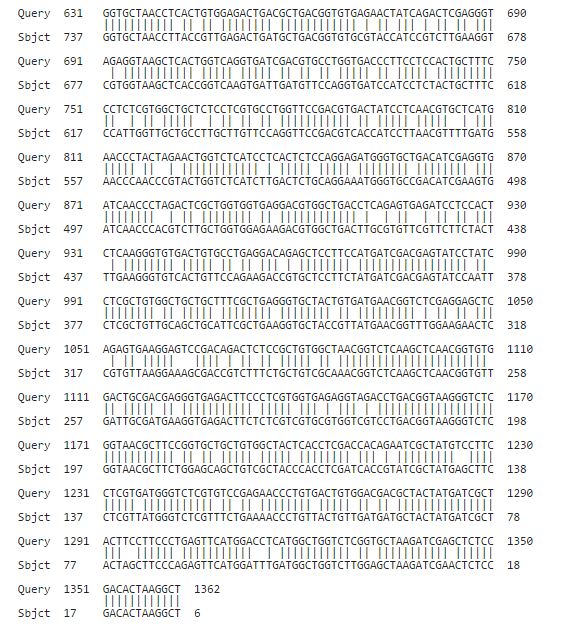

Supplement: Supplementary file 4 — Additional file 4. Comparison of CEMB glyphosate resistant gene vs monsanto glyphosate resistant gene (3/3). [file 13104_2015_1397_MOESM4_ESM.jpeg]
